# A retrospective real-world study on the safety and efficacy of budesonide orodispersible tablets for the induction therapy of eosinophilic oesophagitis

**DOI:** 10.1177/17562848241290346

**Published:** 2024-10-16

**Authors:** Rachel Geow, Gina Arena, Chiang Siah, Sherman Picardo

**Affiliations:** UWA Medical School, The University of Western Australia, Perth, Australia, 17 Monash Avenue, Nedlands 6009, Australia; UWA Medical School, The University of Western Australia, Perth, Australia; Department of Gastroenterology, Royal Perth Hospital, Perth, Australia; Department of Gastroenterology, Royal Perth Hospital, Perth, Australia; Curtin University, Perth, Australia

**Keywords:** budesonide, candida, eosinophilic oesophagitis, gastrointestinal, steroid

## Abstract

**Background:**

An orodispersible form of budesonide has recently been approved for the targeted treatment of eosinophilic oesophagitis in the United Kingdom, Europe, Australia, Canada and the United States, following favourable results from a randomised controlled trial. This is the first dedicated real-world study exploring the safety and efficacy of budesonide orodispersible tablets for induction therapy in the treatment of eosinophilic oesophagitis while providing insights into its management.

**Objectives:**

The primary objective was histologic remission, defined as less than 5 eosinophils per high-powered field. The secondary objectives included histologic response (>50% reduction in peak eosinophil count), clinical remission (complete resolution of symptoms documented on clinic letters), clinical response (improvements in symptoms as reported on clinical letters), endoscopic remission (Endoscopic Reference Score (EREFS) score = 0), and endoscopic response (improvement in EREFS score). The EREFS scores were calculated based on the severity and presence of rings, longitudinal furrows, strictures, oedema and exudates on endoscopic images. Adverse events and safety profiles were also recorded.

**Design:**

A multicentre cohort study examining the effectiveness of 1 mg, twice daily, budesonide orodispersible tablet induction therapy for the treatment of eosinophilic oesophagitis.

**Methods:**

Ethics approval was obtained through the Western Australia Health: Governance, Evidence, Knowledge, Outcomes system for assessment of Audit and Quality Activities. The study adhered to the Strengthening the Reporting of Observational Studies in Epidemiology guidelines.

**Results:**

A total of 43 patients (29 males, 14 females; median age 39) were recruited. Forty-one patients were included in the analysis. After induction therapy, 30 patients (73%) achieved histologic remission, and 35 patients (85%) demonstrated histologic response. Thirty-nine patients (95%) achieved clinical response, and 28 patients (68%) achieved clinical remission. An endoscopic response was seen in 37 patients (90%), and 16 patients (39%) achieved endoscopic remission. No significant adverse events were identified.

**Conclusion:**

Budesonide orodispersible tablet is an effective induction therapy for eosinophilic oesophagitis, as evidenced by its high histologic remission rate and favourable safety profile.

## Introduction

Eosinophilic oesophagitis (EoE) is a chronic inflammatory disease of the oesophagus characterised by symptoms of oesophageal dysfunction and eosinophilia, defined by the presence of more than 15 eosinophils per high-powered field (hpf) on histologic specimens.^1^ There has been a significant increase in the incidence and prevalence of EoE, particularly in Western countries over the last two decades.^2^ Current treatments for EoE include non-pharmacological interventions such as food elimination diets and endoscopic dilation for stricturing disease, and pharmacological treatments such as proton pump inhibitors (PPI), swallowed topical corticosteroids and anti-interleukin-4 receptor biologic, dupilumab, that was recently approved for use in the United States for the treatment of EoE.^1,3^ Swallowed topical corticosteroids are often considered the first-line treatment for managing symptoms and inflammation in active disease, although treatment for EoE has generally involved off-label use of corticosteroid inhalers or compounded slurries.^4^ A phase III randomised controlled trial (RCT) published in 2019 found that an orodispersible formulation of budesonide was effective in inducing remission in patients with EoE.^4^ This study involved 59 patients and achieved clinicohistological remission rates of 58% and 85% following 6 and 12 weeks of treatment, respectively, compared to 0% in the placebo group.^4^ Furthermore, endoscopic remission, characterised by complete normalisation of oesophageal appearance, was achieved in 61% of patients, compared to 0% in the placebo group.^4^ The study also found that budesonide orodispersible tablet (BOT) was well tolerated, with no serious events reported.^4^ A recent meta-analysis comparing pharmaceutical drugs for EoE treatment corroborated the effectiveness of BOT 1 mg, twice daily, particularly in achieving histologic remission.^5^

Following the positive results from the phase III RCT, BOT has been approved for use as induction therapy in patients with EoE in several jurisdictions around the world including the United Kingdom, Europe, Australia and Canada. It has recently been approved for the treatment of EoE in the United States. However, real-world data on the efficacy and safety of BOT remain limited.

This is the first dedicated real-world study, aiming to evaluate the safety and efficacy of BOT, for the treatment of EoE and provide insights into its management.

## Materials and methods

### Study design

A multicentre retrospective cohort study was conducted at two hospitals (Royal Perth Hospital and Bentley Hospital) in Western Australia between July 2022 and March 2023. Ethics approval was obtained through the Western Australia Health: Governance, Evidence, Knowledge, Outcomes (GEKO) system for the assessment of Audit and Quality Activities. The reporting of this study conforms to the Strengthening the Reporting of Observational Studies in Epidemiology (STROBE) statement (Supplemental Material).^6^

### Participants

All patients with a diagnosis of EoE beginning an induction course of BOT 1 mg, twice daily, irrespective of prior therapies, were included in the study. The use of PPI prior to commencing BOT was not a diagnostic requirement for study enrolment. A detailed history of past EoE therapies was not available for all patients; however, all EoE therapies (PPI, corticosteroids and/or dietary) were stopped prior to commencing BOT. The diagnosis of EoE was made based on a combination of clinical symptoms, such as dysphagia, food bolus obstruction and reflux; endoscopic findings of furrows, rings, and strictures and/or oedema and exudates; histopathology showing an eosinophil count more than 15 eosinophils per hpf; and exclusion of other possible cause such as gastroesophageal reflux disease. All patients had an index endoscopy performed prior to treatment initiation and a follow-up endoscopy between 8 and 10 weeks after commencing therapy. Patients who did not complete therapy, or failed to have an end-of-treatment endoscopy, were excluded from the analysis. All patients had clinical appointments prior to and after completing induction therapy. Patient socio-demographics, disease-specific factors, and endoscopic and histologic results were collected from i.ClinicalManager, a hospital patient information system, and clinic letters detailing clinical symptoms. Patient co-morbidities were not obtained due to the retrospective nature of the study.

### Outcomes

Our primary objective was the rate of histologic remission post-induction therapy, defined as less than 5 eosinophils per high-powered field (eosinophils/hpf), which was similar to the definition used in the study by Lucendo et al.^4^ Secondary objectives included the rate of histologic response (>50% reduction in peak eosinophil count), clinical remission (complete resolution of symptoms documented on clinic letters, i.e. no dysphagia, odynophagia, or food obstruction), clinical response (improvements in clinical symptoms as reported on clinic letters, with no episode of food obstruction), endoscopic remission (Endoscopic Reference Score (EREFS) score = 0) and endoscopic response (improvement in EREFS score). The EREFS scores were calculated based on the severity and presence of rings, longitudinal furrows, strictures, oedema and exudates on endoscopic images, which were confirmed and centrally read by an experienced gastroenterologist in EoE. Histologic biopsies were taken by endoscopists at two locations, with at least six biopsies taken cumulatively as per EoE diagnostic protocol. All adverse events and side effects were recorded.

### Statistical analysis

Statistical analyses were performed using Stata.^7^ A descriptive analysis was conducted for continuous variables which were reported as either mean ± SD or median (interquartile range (IQR)) for skewed distributions, while categorical or binary variables were presented as proportions or percentages. The Wilcoxon sign-rank test was used for pairwise comparisons between pre- and post-treatment EREFS scores and eosinophil counts. Significance was defined as a *p* value less than 0.05. Side effects and adverse events were described quantitatively.

## Results

Forty-three participants were included, of which 68% were male and the median age was 39 years. Two patients discontinued therapy due to intolerance prior to the end of the treatment scope and hence were excluded from the analysis. Patient demographics are summarised in Table 1.

**Table 1.** Baseline characteristics.

Characteristics	***n*** = 43
Age, years^a^	39 (30–50)
Sex, male	29 (67%)
Food bolus obstruction	14 (33%)
Index endoscopy	
EREFS score	4 (3–5)
Strictures	13 (30%)
Peak **eosinophil count/HPF**^a^	45 (34–60)

a Expressed as median (interquartile range).

EREFS, Endoscopic Reference Score; HPF, high-powered field.

### Remission and response

**Figure 1.**
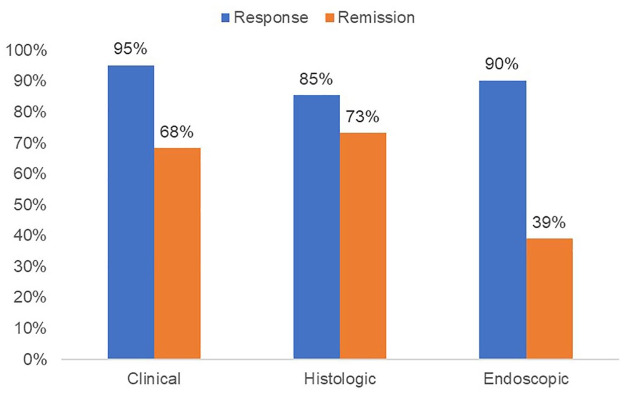
Efficacy of BOT for EoE after 8–10 weeks of treatment (post-analysis) for each of clinical, histologic, and endoscopic remission and response. BOT, budesonide orodispersible tablet; EoE, eosinophilic oesophagitis.

**Figure 2.**
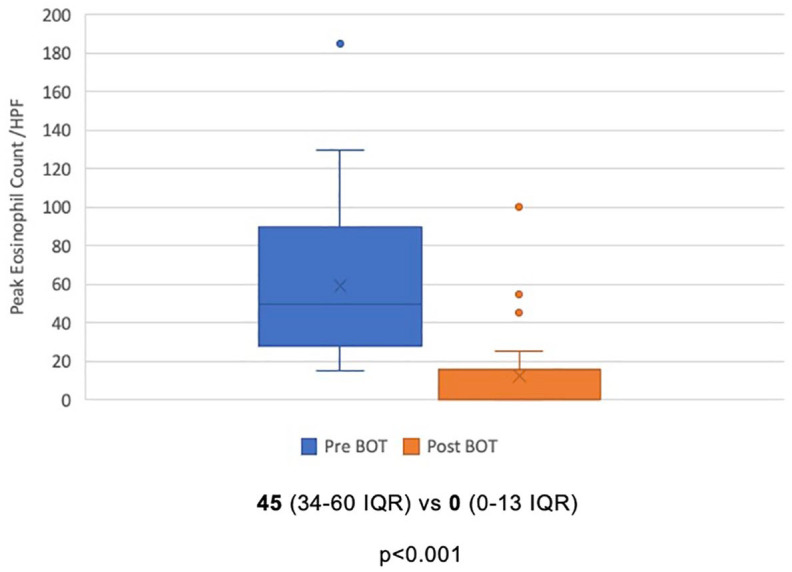
Mean peak eosinophil count before and after treatment with BOT. Expressed as a mean ± interquartile range. BOT, budesonide orodispersible tablet.

**Figure 3.**
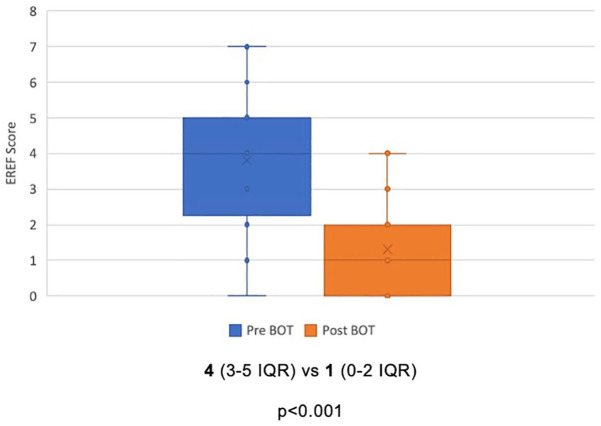
EREFS score before and after treatment with BOT. Expressed as a mean ± interquartile range. BOT, budesonide orodispersible tablet; EREFS, Endoscopic Reference Score.

Among 41 patients included in the analysis, a total of 30 patients (73%) achieved histologic remission after BOT induction therapy. Clinical and endoscopic remission rates were 68% and 39%, respectively. Ninety-five percent of patients had a clinical response, while 85% demonstrated a histologic response, and 90% achieved an endoscopic response (Figure 1). There was a statistically significant reduction in peak eosinophil count, from a median of 45/hpf (34–60 IQR) to 0/hpf (0–13 IQR) after induction therapy (*p* < 0.001) (Figure 2). Similarly, EREFS scores improved from a median of 4 (3–5 IQR) to 1 (0–2 IQR) at the end of treatment (*p* < 0.001) (Figure 3).

### Adverse events and side effects

Out of the 43 participants included in the study, a total of two patients (4.7%) had discontinued therapy prior to the end of induction due to side effects. One had a sensation of tongue and throat swelling with no abnormalities identified on clinical examination. The second reported mild abdominal discomfort as well as a lack of perceived efficacy with therapy. Out of 41 patients who completed induction, side effects (sore throat, nausea, abdominal pain, tongue blistering) were reported by three patients (7.3%). A further three patients (7.3%) were noted to have candida on oesophageal biopsies, which were managed with antifungal therapy.

## Discussion

Eosinophilic oesophagitis is an increasingly prevalent condition worldwide with limited treatment options available at present.^8,9^ In this multicentre real-world study, we demonstrate the efficacy of BOT for the induction therapy of EoE with a histologic remission rate of 73% and excellent response rates in clinical and endoscopic domains. Treatment with BOT also had a favourable safety profile with minimal reported adverse events. The results of our study are comparable to the phase III randomised placebo-controlled clinical trial by Lucendo et al.^4^ In this study, 57.6% of patients receiving BOT therapy achieved the primary endpoint, a combination of clinical and histologic remission at week 6, compared to 0% in the placebo group.^4^ Histologic remission was achieved in 93.2% of patients, clinical remission in 59.3% and endoscopic remission in 61%.^4^ Our study demonstrated lower rates of histologic remission at 73%, clinical remission at 68% and endoscopic remission at 39%, likely reflecting real-world clinical practice, influenced by factors such as a more diverse patient population, co-morbidities and compliance with medication. Our cohort also had a higher baseline burden of disease as compared with the phase III trial with higher rates of stricture (30% vs 15%) as well as a higher median EREFS score.^4^ If using the less stringent and more widely accepted remission criteria of less than 15 eosinophils/hpf, 78% of our patients achieved this outcome following induction therapy.^4^

Our study is the first dedicated real-world study evaluating BOT therapy for the treatment of EoE. A recent registry study published by Laserna-Mendieta et al. reported on the efficacy of various formulations of swallowed corticosteroids for the treatment of EoE.^10^ This included some patients exposed to BOT therapy, either 1 or 2 mg daily, with varying follow-up intervals.^10^ They demonstrated a combined clinicohistological remission rate of 94%.^10^ There have also been multiple studies looking at other steroid formulations for the treatment of EoE which yielded mixed results.^11–13^ A 2012 RCT on aerolised fluticasone demonstrated poor results, where a non-significant rate of clinical remission was found, despite a 62% histologic response rate.^12^ This was likely due to an underpowered study with a small sample size (*n* = 21), high drop-out rates and changes to biopsy protocols during the study.^12^ In a comparative study evaluating the histologic response in patients with EoE treated with budesonide slurry and fluticasone inhaler, the study found that budesonide slurry was slightly superior in achieving histologic response (<15 eosinophils/hpf), with 71% of patients achieving histologic response compared to 64% in the fluticasone inhaler group.^11^ However, budesonide slurry was not statistically superior following analysis.^11^ The variability in dosage and administration of budesonide slurry and fluticasone inhalers may have affected the outcomes of the study.

In a recent phase IIb clinical study assessing the efficacy of fluticasone propionate oral disintegrating tablets across three dosage levels, histologic response (<6 eosinophils/hpf) was observed ranging between 48% and 86% of patients, depending on dose following 12 weeks of treatment.^13^ These rates appeared lower than the findings reported in the study evaluating BOT therapy over a 6-week period, where histologic remission (<5 eosinophils/hpf) was achieved in 93.2% of patients.^4,13^

A phase III study investigating the efficacy of budesonide oral suspension for induction therapy over 12 weeks found that the treatment demonstrated effectiveness compared to placebo for the treatment of EoE.^14^ This study, however, had lower absolute histologic remission rates (53.1%) and clinical response rates (52.6%), compared to the BOT phase III and our real-world study.^4^ Unlike the orodispersible tablet, which leverages saliva to enhance the contact time of budesonide with the oesophagus, the oral suspension may lack this extended contact period.^15^ Treatment with BOT demonstrates the best response rates compared to all other steroid formulations evaluated in EoE.

Oral candidiasis is a commonly reported side effect of budesonide treatment and was found to occur at similar frequencies in multiple studies.^4,11,14^ Amongst patients taking BOT, Lucendo et al. reported candidiasis in 16.9% of patients, whilst 7.1% of patients were found to have oesophageal candida.^4^ The frequency of oesophageal candidiasis in patients on budesonide slurry was found to range from 3.8% to 12%.^11,14^ Amongst patients on budesonide treatment, most had mild candidiasis and were easily treated with antifungals.^4,11,14^ Overall, budesonide therapy demonstrates a good safety profile despite the incidence of oesophageal candidiasis. The cases of oesophageal candidiasis were usually mild and readily treatable and hence did not compromise the overall safety of budesonide therapy.

Currently, the universally accepted criterion for diagnosing EoE is a histologic examination showing at least 15 eosinophils/hpf, with or without clinical symptoms.^16^ There were five patients (12%) in our study who achieved a histologic response but not remission, of which four also achieved a clinical response. Thirty-two patients (78%) achieved a post-treatment peak eosinophil count of less than 15 eosinophils per high-powered field, the traditional endpoint for histologic remission.

The main advantage of our study includes our robust data collection method, where endoscopic, clinical and histologic results were obtained simultaneously. Histology was also read by a specialised gastrointestinal pathologist. We employed a validated endoscopic scoring tool – EREFS, with an experienced central reader scoring endoscopic images of study participants, which increases the internal validity of our study.

### Limitations

There were several limitations to our study. Firstly, due to the nature of a retrospective study, we were unable to control variables or treatment protocols that had lapsed. As such, we were unable to objectively assess clinical symptoms as patients were not provided with a symptom questionnaire before and after BOT therapy.

Secondly, our study involved different clinicians performing endoscopies for patients. Having different clinicians perform the procedures could lead to variations in how endoscopic features were assessed and where biopsies were taken. A 2021 Danish study has recommended that four oesophageal biopsies should be taken following the ‘4-14-4 rule’, where biopsies should be taken from 4 and 14 cm above the gastroesophageal junction.^17^ This rule aims to prevent underestimation of the true peak eosinophil count, which can contribute to the underdiagnosis of EoE.^17^ Unfortunately, in our study, we were not able to ascertain the actual locations from which biopsies were taken, hence promoting information bias and affecting the accuracy and reliability of our findings. The other variable factor was the timing of when endoscopies were performed. In our study, patients were able to have their end-of-treatment scope between 8 and 10 weeks. This meant that the actual rate of outcomes across the various domains could be underestimated, as a longer duration of treatment may lead to higher response and remission rates.

Thirdly, our study did not assess compliance with medications as patients were only reviewed at the start and end of treatment. This could confound the actual rates of response and remission across the three domains, as medication non-compliance could similarly affect treatment outcomes.

## Conclusion

In summary, we report that BOT is an effective induction therapy for EoE, with a 73% rate of histologic remission. Budesonide orodispersible tablet is also a relatively safe drug, with minimal side effects, the most common being oesophageal candidiasis. It also demonstrates the best clinical and histological response rates compared to all other steroid therapies that have been evaluated for the treatment of EoE. Further research should aim to determine a treatment response criterion and investigate an extended induction duration of BOT therapy for certain patient populations.

## Supplemental Material

sj-pdf-1-tag-10.1177_17562848241290346 – Supplemental material for A retrospective real-world study on the safety and efficacy of budesonide orodispersible tablets for the induction therapy of eosinophilic oesophagitis Supplemental material, sj-pdf-1-tag-10.1177_17562848241290346 for A retrospective real-world study on the safety and efficacy of budesonide orodispersible tablets for the induction therapy of eosinophilic oesophagitis by Rachel Geow, Gina Arena, Chiang Siah and Sherman Picardo in Therapeutic Advances in Gastroenterology

## Declarations

**Ethics approval and consent to participate:** Royal Perth Bentley Group – GEKO (Governance Evidence Knowledge Outcomes) Quality Activity 47821. Approval date: 14-Nov-2022.

**Consent for publication:** Not applicable.

**Availability of data and materials:** The data that support the findings of this study are available from the corresponding author, R.G, upon reasonable request.

## References

[1] Chang JW Kliewer K Haller E , et al. Development of a practical guide to implement and monitor diet therapy for eosinophilic esophagitis. Clin Gastroenterol Hepatol 2023; 21: 1690–1698. 36933603 10.1016/j.cgh.2023.03.006 PMC10293042

[2] Hahn JW Lee K Shin JI , et al. Global incidence and prevalence of eosinophilic esophagitis, 1976–2022: a systematic review and meta-analysis. Clin Gastroenterol Hepatol 2023; 21: 3270–3284.e77. 10.1016/j.cgh.2023.06.005 37331411

[3] Greuter T Hirano I Dellon ES. Emerging therapies for eosinophilic esophagitis. J Allergy Clin Immunol 2020; 145: 38–45. 31705907 10.1016/j.jaci.2019.10.027 PMC6981295

[4] Lucendo AJ Miehlke S Schlag C , et al. Efficacy of budesonide orodispersible tablets as induction therapy for eosinophilic esophagitis in a randomized placebo-controlled trial. Gastroenterology 2019; 157: 74–86.e15. 10.1053/j.gastro.2019.03.025 30922997

[5] Visaggi P Barberio B Del Corso G , et al. Comparison of drugs for active eosinophilic oesophagitis: systematic review and network meta-analysis. Gut 2023; 72: 2019–2030. 37491157 10.1136/gutjnl-2023-329873

[6] von Elm E Altman DG Egger M , et al. The Strengthening the Reporting of Observational Studies in Epidemiology (STROBE) statement: guidelines for reporting observational studies. Lancet 2007; 370: 1453–1457. 18064739 10.1016/S0140-6736(07)61602-X

[7] StataCorp. Stata statistical software (Version 17) [Computer software]. College Station, TX: StataCorp LLC, 2021.

[8] Dellon ES Hirano I. Epidemiology and natural history of eosinophilic esophagitis. Gastroenterology 2018; 154: 319–332.e3. 10.1053/j.gastro.2017.06.067 PMC5794619 28774845

[9] Roberts SE Morrison-Rees S Thapar N , et al. Incidence and prevalence of eosinophilic oesophagitis across Europe: a systematic review and meta-analysis. United European Gastroenterol J 2024; 12(1):89-102. 10.1002/ueg2.12465 PMC10859717 37921701

[10] Laserna-Mendieta EJ Navarro P Casabona-Francés S , et al. Swallowed topical corticosteroids for eosinophilic esophagitis: utilization and real-world efficacy from the EoE CONNECT registry. United European Gastroenterol J 2024; 12: 585–595. 10.1002/ueg2.12533 PMC11176909 38284792

[11] Dellon ES Woosley JT Arrington A , et al. Efficacy of budesonide vs fluticasone for initial treatment of eosinophilic esophagitis in a randomized controlled trial. Gastroenterology 2019; 157: 65. 30872104 10.1053/j.gastro.2019.03.014 PMC6581596

[12] Alexander JA Jung KW Arora AS , et al. Swallowed fluticasone improves histologic but not symptomatic response of adults with eosinophilic esophagitis. Clin Gastroenterol Hepatol 2012; 10: 742–749.e1. 10.1016/j.cgh.2012.03.018 22475741

[13] Dellon ES Lucendo AJ Schlag C , et al. Fluticasone propionate orally disintegrating tablet (APT-1011) for eosinophilic esophagitis: randomized controlled trial. Clin Gastroenterol Hepatol 2022; 20: 2485–2494.e15. 10.1016/j.cgh.2022.02.013 35181572

[14] Hirano I Collins MH Katzka DA , et al. Budesonide oral suspension improves outcomes in patients with eosinophilic esophagitis: results from a phase 3 trial. Clin Gastroenterol Hepatol 2022; 20: 525–534.e10. 10.1016/j.cgh.2021.04.022 33887475

[15] Feo-Ortega S Lucendo AJ. Evidence-based treatments for eosinophilic esophagitis: insights for the clinician. Therap Adv Gastroenterol 2022; 15: 17562848211068665. 35069803 10.1177/17562848211068665 PMC8777364

[16] Rawla P Sunkara T Thandra KC , et al. Efficacy and safety of budesonide in the treatment of eosinophilic esophagitis: updated systematic review and meta-analysis of randomized and non-randomized studies. Drugs R D 2018; 18: 259–269. 30387081 10.1007/s40268-018-0253-9 PMC6277325

[17] Krarup AL Drewes AM Ejstrud P , et al. Implementation of a biopsy protocol to improve detection of esophageal eosinophilia: a Danish registry-based study. Endoscopy 2021; 53: 15–24. 32757199 10.1055/a-1206-0852

